# Quantification of Smoking Characteristics Using Smartwatch Technology: Pilot Feasibility Study of New Technology

**DOI:** 10.2196/20464

**Published:** 2021-02-05

**Authors:** Casey Anne Cole, Shannon Powers, Rachel L Tomko, Brett Froeliger, Homayoun Valafar

**Affiliations:** 1 Department of Computer Science and Engineering University of South Carolina Columbia, SC United States; 2 Department of Psychological Sciences University of Missouri-Columbia Columbia, MO United States; 3 Department of Psychology University of Denver Denver, CO United States; 4 Department of Psychiatry & Behavioral Sciences Medical University of South Carolina Charleston, SC United States; 5 Department of Psychiatry University of Missouri-Columbia Columbia, MO United States

**Keywords:** smartwatch, CReSS, smoking topography, ASPIRE, automated, wearable technology, wearable computing, smoking

## Abstract

**Background:**

While there have been many technological advances in studying the neurobiological and clinical basis of tobacco use disorder and nicotine addiction, there have been relatively minor advances in technologies for monitoring, characterizing, and intervening to prevent smoking in real time. Better understanding of real-time smoking behavior can be helpful in numerous applications without the burden and recall bias associated with self-report.

**Objective:**

The goal of this study was to test the validity of using a smartwatch to advance the study of temporal patterns and characteristics of smoking in a controlled laboratory setting prior to its implementation in situ. Specifically, the aim was to compare smoking characteristics recorded by Automated Smoking PerceptIon and REcording (ASPIRE) on a smartwatch with the pocket Clinical Research Support System (CReSS) topography device, using video observation as the gold standard.

**Methods:**

Adult smokers (N=27) engaged in a video-recorded laboratory smoking task using the pocket CReSS while also wearing a Polar M600 smartwatch. In-house software, ASPIRE, was used to record accelerometer data to identify the duration of puffs and interpuff intervals (IPIs). The recorded sessions from CReSS and ASPIRE were manually annotated to assess smoking topography. Agreement between CReSS-recorded and ASPIRE-recorded smoking behavior was compared.

**Results:**

ASPIRE produced more consistent number of puffs and IPI durations relative to CReSS, when comparing both methods to visual puff count. In addition, CReSS recordings reported many implausible measurements in the order of milliseconds. After filtering implausible data recorded from CReSS, ASPIRE and CReSS produced consistent results for puff duration (*R^2^*=.79) and IPIs (*R^2^*=.73).

**Conclusions:**

Agreement between ASPIRE and other indicators of smoking characteristics was high, suggesting that the use of ASPIRE is a viable method of passively characterizing smoking behavior. Moreover, ASPIRE was more accurate than CReSS for measuring puffs and IPIs. Results from this study provide the foundation for future utilization of ASPIRE to passively and accurately monitor and quantify smoking behavior in situ.

## Introduction

Tobacco use disorder (TUD) is the leading preventable cause of death worldwide (including the United States) [1], and the costs associated with its treatment and prevention remain a major economic burden on society [2]. Therefore, a better understanding of the behavioral elements and mechanisms that maintain smoking behavior is critically important for preventing future smoking-related illnesses. While there have been substantial technological advances in studying the neurobiological and clinical bases of TUD and nicotine addiction, there have been relatively minor advances in technologies for monitoring, characterizing, and intervening on smoking behavior. Therefore, there is a critical need for leveraging emerging technologies that may help provide personalized strategies for smoking cessation.

Traditional approaches to the study of human behavior primarily rely upon self-reporting or laboratory observations. Despite strengths found in self-report and laboratory-based research, those techniques, by design, are prone to limitations in external validity and are subject to human error. To more fully and accurately characterize and understand factors influencing people’s behavior, enabling technologies must be developed to allow nonintrusive and longitudinal observation of human behavior in natural settings. There are numerous advantages in passive and accurate characterization of smoking in real time with temporal precision [3] and without recall biases. Real-time quantification of smoking duration before and during a cessation attempt can help to develop a more personalized and effective cessation protocol. Wearable devices are well-positioned to passively assess a person's behavior in the aforementioned context.

Wearable devices are equipped with a rich array of sensors (accelerometer, gyroscope, magnetometer, barometer, GPS, heart rate, electrocardiogram, oximeter) and may serve as a powerful platform for nonintrusively capturing and studying human behavior. In addition, the availability of mobile and wearable devices is an international phenomenon, and their use is not confined to any particular socioeconomic class. Therefore, the use of these devices for sensing, recording, and identifying human activities has the potential to passively observe health behaviors and be deployed internationally without confinement by any socioeconomic, political, or geographical barriers.

In recent years, there have been several reports of utilizing commercially available smartwatches in studying human activities. These include generic activities such as step counts, sleep detection, and rest periods, while others include more specific activities such as eating [4], drinking [5], managing diabetes [6,7], or smoking [8]. Previous work has established the use of wristworn devices in observing and interpreting smoking behavior in laboratory settings [9-13] and in situ [8,14]. Some of these devices use proprietary sensors [5,15-17], while others use off-the-shelf devices such as smartwatches [8,9,14,18,19]. The use of smartwatches in continuous monitoring of human activities and behavior is compelling for several reasons including their availability, decreasing cost, popularity, and the convenience and completeness of data collection. The data connectivity that is afforded by smartwatches adds a critical component to their appeal, allowing for real-time observation and interpretation of activities that can lead to immediate deployment of the appropriate intervention. Artificial intelligence (AI)-assisted detection of smoking with smartwatch technology [8-10,14,18-20] can reduce the burden of self-reporting by the user and automate notification of the team of research scientists and the caregivers. Despite their potential, the accuracy and resolution of data collected by smartwatches have not been comprehensively explored in comparison to traditional smoking behavior assessments. More specifically, while it has been shown that detection of smoking a cigarette is possible with a smartwatch [8,9,14,18,21,22], the use of smartwatches in better exploring more detailed smoking characteristics of human subjects remains unanswered. Smoking characteristics (eg, number of puffs, the length of smoking session, duration of puffs) vary across smokers, are associated with overall toxicant exposure [23], and are subject to modification with smoking cessation pharmacotherapy [24]. Therefore, accurate quantification of smoking characteristics using a smartwatch in naturalistic settings is advantageous in order to estimate smokers’ toxicant exposure in daily life.

Observation of smoking using smartwatches has several compelling aspects. First, smartwatches have sufficient storage capacity to record and store sensor data for a duration longer than 24 hours. The recording duration can be extended into months by the addition of micro-SD storage media to the companion phone. Second, the collected data will require no additional action (other than periodic charging of the device) by the user or the participant of a study, qualifying this method of data acquisition to be highly unobtrusive. Third, unlike self-reporting approaches, continuous recording of sensor data can provide a comprehensive report of a person’s activities in natural settings prior to and after the event of interest. For instance, proper interpretation of the sensor data can provide a detailed view of related human activities such as drinking, eating, sleeping, exercising, and smoking all in one experiment. The collection of such a detailed ensemble of activities is nearly infeasible through self-reporting when observed in situ. Fourth, the real-time connectivity of smartwatches allows for real-time observation of human behavior, which can be used in numerous ways to study or augment human behavior. For example, the adherence of a subject to study protocols can be viewed and confirmed, and if necessary, notifications and reminders can be sent to the participants. Real-time and continuous connectivity with participants allows for the initiation of the appropriate actions, paving the way for personalized intervention or cessation approaches.

In this report, we present an evaluation and comparison of the quality of smoking data collected by smartwatches, Clinical Research Support System (CReSS), and video recordings of participants in a laboratory setting. In particular, we compared and contrasted the accuracy of observing interpuff intervals (IPIs) and puff duration (PD) using the Automated Smoking PerceptIon and REcording (ASPIRE) smartwatch application and the CReSS device. We resorted to human annotation of visual recordings of smoking sessions when possible to resolve substantial disagreements between the ASPIRE and CReSS approaches. We also explored the additional capabilities of the ASPIRE-based method and comment on the significant advantages that it affords and its novel future utilities.

## Methods

### CReSS Device

The CReSS Pocket (Borgwaldt KC Inc, Richmond, VA) is widely used for studying smoking in a laboratory setting [25-28] and is considered the gold standard of data collection in the field of smoking research. Though the CReSS device provides objective measures of smoking topography and is amenable to use in a laboratory setting, it is relatively expensive (US ~$5500) and interferes with the natural smoking experience. These issues limit its utility for characterizing ad lib smoking in a smoker’s natural environment, while interfering with normal smoking patterns in a laboratory.

### Characterization of Smoking Topography Using a Smartwatch

Smoking topography has been used to refer to a number of specific aspects of smoking behavior such as puff volume, maximum puff velocity, IPI, PD, number of puffs per cigarette, and total smoking duration.[25] In this study, we adopted a condensed set of smoking characteristics, namely IPI and PD, as the topography of smoking. These 2 characteristics (IPI and PD) can be used to calculate nearly all of the remaining measures such as the total duration of smoking, puff velocity (and therefore maximum, minimum, and medial puff velocity), and number of puffs per smoking session (or a cigarette). However, the measure of puff volume is the only parameter that cannot be directly calculated from the accelerometer data.

A smartwatch-based method allows participants to smoke freely in their natural settings without the need to use an intermediary device. Our previous work reported the development of an Android Wear OS-based software (ASPIRE) package that is capable of recording [9] and automating detection of smoking gestures (puff) [14]. ASPIRE incorporates a hierarchy of AI techniques in order to achieve automated detection of smoking sessions with as high as 97% success in laboratory settings [9,18] and 90% success in natural settings [14]. Previous work has established the high accuracy of ASPIRE in detection of smoking sessions, but its performance in the quantification of detailed smoking characteristics has not been reported. The more fine-grained assessment of a smoking session is clearly a more challenging task and provides useful information that can be invaluable in efforts to develop personalized cessation plans.

In the current study, participants were provided a smartwatch (Polar M600) and Android smartphone for the duration of data collection. Our selection of the smartwatch (Polar M600) primarily was based on a balance between the cost of the equipment (limited to US $150), availability of the needed sensors (accelerometer and gyroscope), programmability using the common Android Studio framework, battery life that exceeded 1 day of use, and Wear OS compatibility. The ASPIRE app listens and collects data from the participant and then sends the data via a Bluetooth connection to the companion smartphone app. The smartphone then uploads the data to a secure server for storage and analysis. The current version of ASPIRE can be obtained from the corresponding author and installed on either a phone/watch pair or as a standalone app on the watch. ASPIRE can be deployed on any smartwatch that is Wear OS compatible.

### Data Collection Protocol

**Figure 1:**
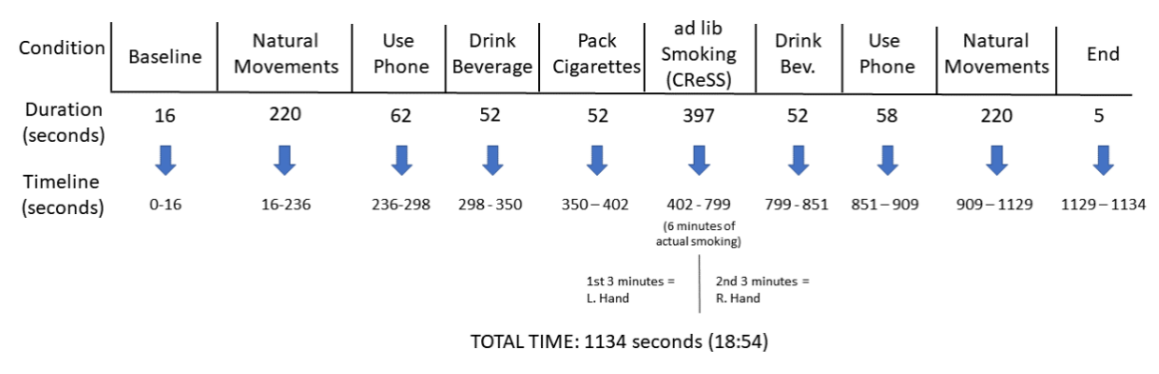
Outline of the protocol used for data collection in the laboratory. CReSS: Clinical Research Support System.

Participants were first outfitted with a Polar M600 to wear on their left hand. Participants were asked to follow a prompt screen on a computer in the laboratory that gave them precise instructions on what behaviors to perform as well as how long to execute each behavior. An overall view of the experimental paradigm with associated durations is shown in Figure 1. For this study, participants were asked to smoke a total of 6 minutes, in which they were asked to split evenly between their left and right hands. In addition to smoking, they were also asked to record over 7 minutes of other movements including 52 seconds of “packing” their cigarette package. It is important to note that the experimental protocol defined here was designed to address a number of questions in a single recording session to optimize the use of human subjects. Some of the targeted investigations in this experiment included the difference in smoking gestures when recorded from left versus right hands, the ability to detect smoking-related activities from the nonsensor hand (the hand without the smartwatch), and recording of other gestures such as packing and opening of a cigarette pack. In addition to recognition of smoking-related activities, other psychological and cognitive parameters related to smoking were measured for other investigations. In this study, we only utilized data related to quantification of IPI and PD.

The CReSS device was used to record the following measures: puff volume, average flow, peak flow, time of peak flow, PD, and IPI. The two measures of interest for this study were PD and IPI. The PD is the length of time in milliseconds that a person inhales for a given intake. The IPI is the number of milliseconds between the end of one puff and the beginning of the next. CReSS records both a high-level and detailed view of these measures. A median measure is used for the high-level view due to the small sample size and existence of outliers that are inherent to the device. The detailed view contains information about each one of the measures per puff. In addition to the data collected by the CReSS and smartwatch devices, videos of each session were recorded and annotated by 2 independent raters (interrater reliability=1.0).

### Participants

Participants were recruited via community advertisements, attended an in-person screening visit to determine eligibility, and then attended an experimental smoking visit. Participants gave written informed consent approved by the Medical University of South Carolina Institutional Review Board and received financial compensation for study participation. Inclusion criteria were age ≥18 years, having an expired carbon monoxide concentration ≥6 ppm (to confirm smoking status), and being willing and able to comply with protocol requirements. The participants (N=35; 13 women, 22 men) were an average age of 43.91 years (SD 12.76 years) with a mean carbon monoxide level of 26.57 ppm (SD 12.32 ppm). Due to recording errors, 8 participants had incomplete CReSS (5/35) or ASPIRE (3/35) data, resulting in a final analytic sample of 27 participants. The primary source of recording errors was deviation from the study protocol.

### Data Annotation Procedure

**Figure 2:**
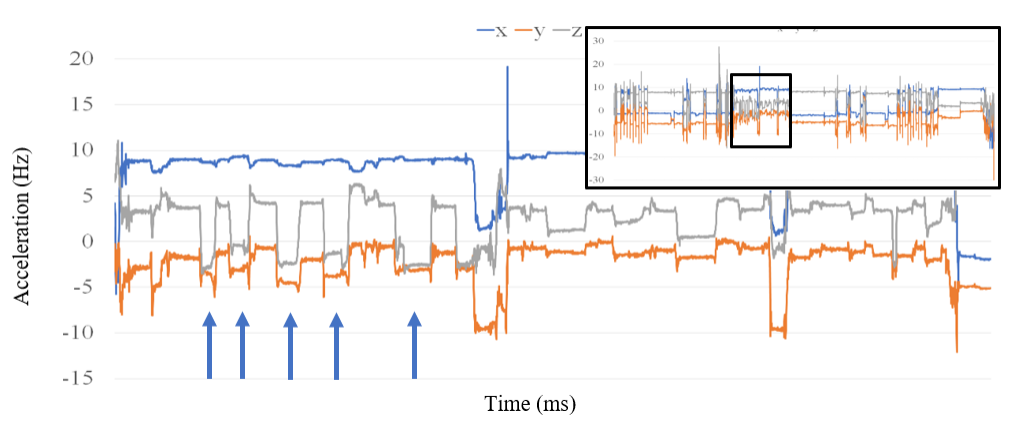
A sample of Automated Smoking PerceptIon and REcording (ASPIRE)'s recording session illustrated in the upper right corner. The main and larger figure depicts the portion of the image that corresponds to a smoking session (arrows indicate the start of a puff).

The first step in the evaluation procedure was to annotate the data collected from smartwatch, CReSS, and video recordings. Due to the time and effort required for the video recordings, only the puff count from each hand was visually enumerated. The annotation for the smartwatch and CReSS device consisted of a well-trained researcher marking the timestamp associated with the beginning and end of each puff. Using this information, PD and IPI were measured. Each puff is easily identifiable as first starting with a slight or negligible change in the x dimension (±1), a moderate decrease in the y dimension (–4), and a sharp decrease in the z dimension (–8) from a resting position (as shown in Figure 2). This is then followed by a period of uninterrupted and equilibrated values of x, y, and z at around 9 m/s^2^, –5 m/s^2^, and –3 m/s^2^, respectively. The end of a smoking gesture was marked as the return of the x, y, and z values to a “resting” state. These numbers vary per participant but will follow the same general pattern.

### Evaluation and Exclusion of Data

The first step in the evaluation of this work was to annotate the data collected from the participants’ puffs. The PDs and IPIs were calculated using these annotations. In the case of ASPIRE, the known sampling rate of 30 Hz was used to convert the timesteps into millisecond units. Figure 2 shows an example of a full set of data (shown in the upper right box) recorded by ASPIRE and a subsection of the data that contained smoking (annotated puffs are denoted with arrows). The period of smoking shown in Figure 2 accounts for approximately 3 minutes of data.

The second phase of the evaluation consisted of calculating the Pearson correlation coefficients between the extracted PDs and IPIs reported by CReSS and ASPIRE. Correlations were first examined using the reported median data for all subjects and then followed by analysis of the data for each individual subject.

Due to the switching of the smoking hand without the transfer of the smartwatch, half of the smoking session was not recorded by the smartwatch. Therefore, using the video recordings of the smoking sessions, we limited our comparison exercise to the portion of the smoking sessions that was recorded by both CReSS and ASPIRE. In addition, in some instances, CReSS reported implausible measures, inclusion of which would provide an inaccurate comparison of the 3 methods. For instance, CReSS reported puffs with a duration of 5 milliseconds or IPIs of >1 minute at the beginning of each smoking session. The long IPI at the beginning of the smoking session is the time the device was turned on to the time of the first puff and was therefore removed from our analysis. The implausibly short puffs reported by CReSS can be explained by a participant performing rapid and multiple puffs such that neither ASPIRE nor the video recordings could identify them. In such instances, we report results with and without the included implausible data since they serve as clear demonstration of some nuances of the CReSS device.

## Results

### Overview of the Collected Data

**Figure 3:**
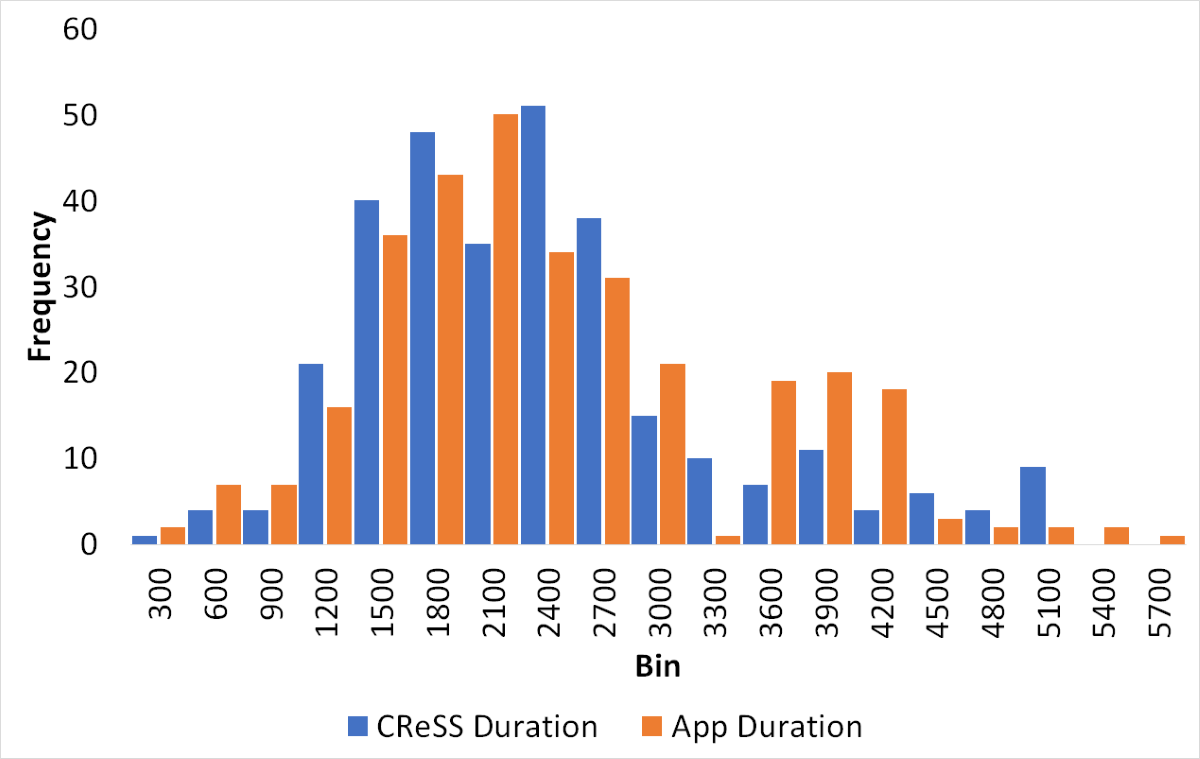
Comparison of individual puff durations collected via the Clinical Research Support System (CReSS; blue) and Automated Smoking PerceptIon and REcording (ASPIRE; orange).

**Figure 4:**
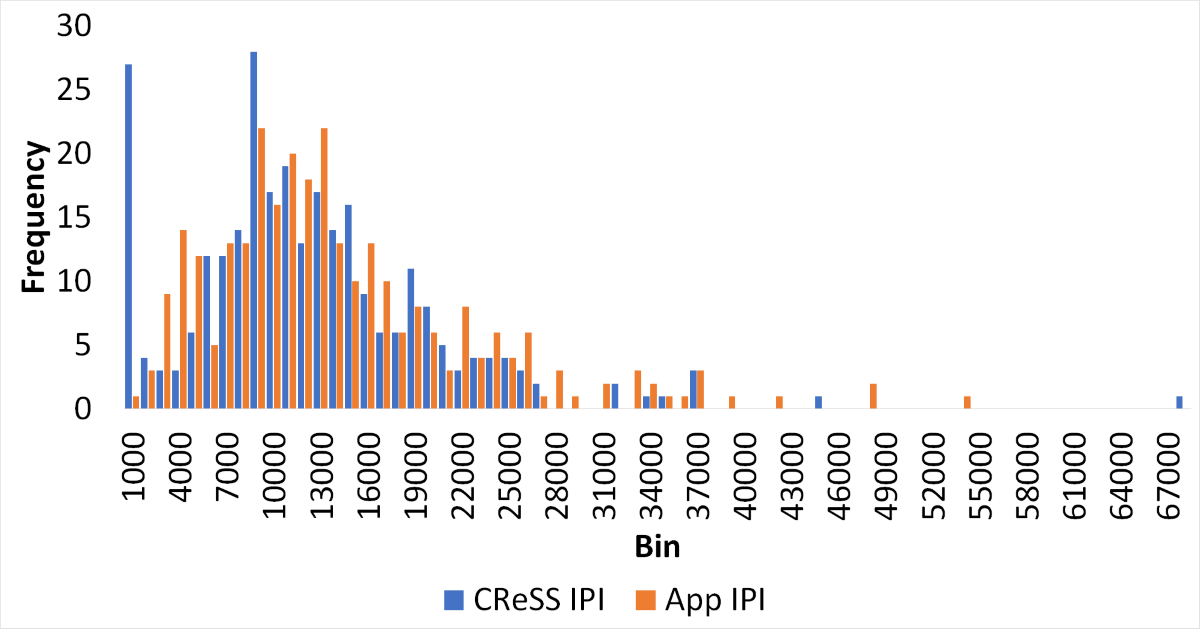
Comparison of individual interpuff intervals (IPIs) collected via Clinical Research Support System (CReSS; blue) and Automated Smoking PerceptIon and REcording (ASPIRE; orange).

Each accelerometer data file collected by ASPIRE contained 20 minutes of data, which is consistent with the experimental protocol. As a first step in our comparison of the 2 methods, histograms were created for individual PDs and IPIs aggregated across all subjects (shown in Figure 3 and Figure 4). The blue bars in these figures correspond to the values produced by the CReSS device, and the orange bars correspond to the values produced by ASPIRE. Although the distributions of the PDs were very similar between the 2 methods, the distributions of IPI values were different (Figure 4). While the 2 histograms demonstrate general agreement, they differ notably in reporting the number of small IPIs. For the CReSS data, there is a spike in very low values corresponding to an IPI value of 0-1 second. The median of the IPIs reported by CReSS in this range was 0.33 seconds.

### Comparison of Overall Statistics

**Figure 5:**
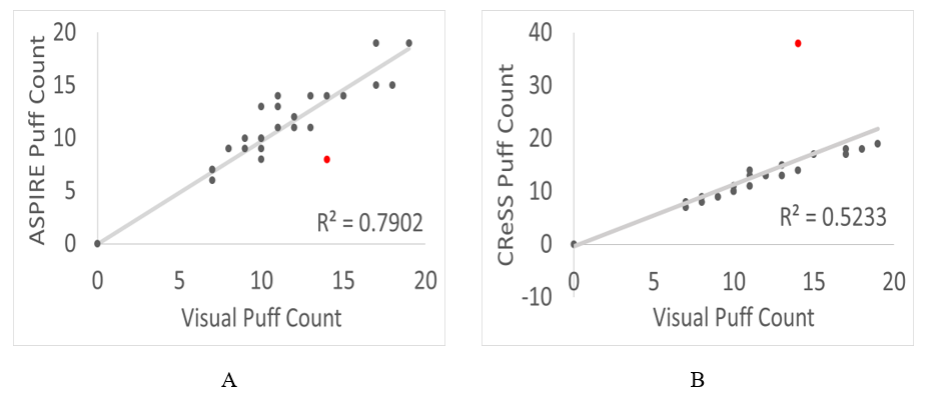
Comparison of the visual puff count versus the (A) Automated Smoking PerceptIon and REcording (ASPIRE) puff count and (B) Clinical Research Support System (CReSS) puff count. In both figures, participant P14 was an outlier and is colored red.

The number of puffs that the CReSS device recorded was compared to a visual inspection of each participant’s respective video recordings. In 80% of cases, the visual puff count and the count reported by CReSS were within ±2. However, in 4 participants, the puff counts differed by as much as ±6. Figure 5A and Figure 5B show the correlations between the overall visual puff counts for the left hand of each participant compared to the puff counts reported by ASPIRE and CReSS, respectively. The R^2^ value for the visual puff count and counts reported by ASPIRE was 0.79, whereas the R^2^ between the visual puff count and the CReSS reported count was 0.52. The participant that caused the most deviation in both comparisons was P14 (in red in Figure 5A and Figure 5B) with reported visual, ASPIRE, and CReSS counts of 14, 8, and 38, respectively.

**Figure 6:**
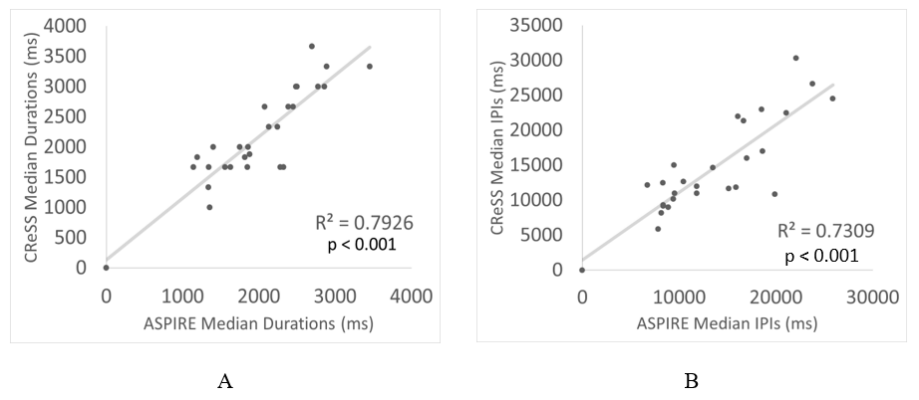
Comparison of the overall reported median (A) Clinical Research Support System (CReSS) versus Automated Smoking PerceptIon and REcording (ASPIRE) puff duration and (B) CReSS versus ASPIRE interpuff interval (IPI).

The correlations between the CReSS and ASPIRE data for the median PD and median IPI across all patients are illustrated in Figure 6A and Figure 6B, respectively. R^2^ values of 0.7926 and 0.7309 (*P*<.001) were calculated for the median PDs and median IPIs, respectively, indicating a high level of correlation between the data reported by the 2 methods.

### Comparison of Individual Puffs

**Figure 7:**
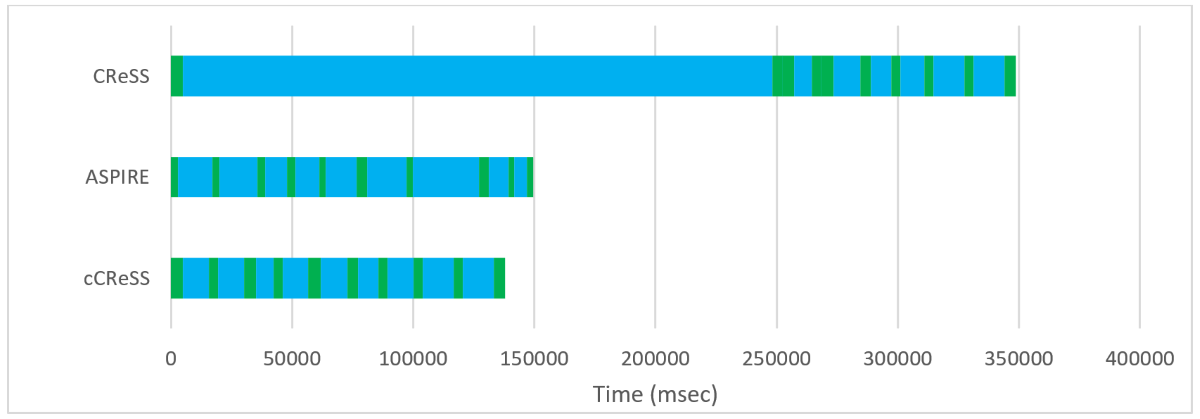
An illustration of smoking topography reported by Clinical Research Support System (CReSS), Automated Smoking PerceptIon and REcording (ASPIRE), and corrected CReSS (cCReSS). The puff durations and interpuff intervals are illustrated in green and blue, respectively.

**Figure 8:**
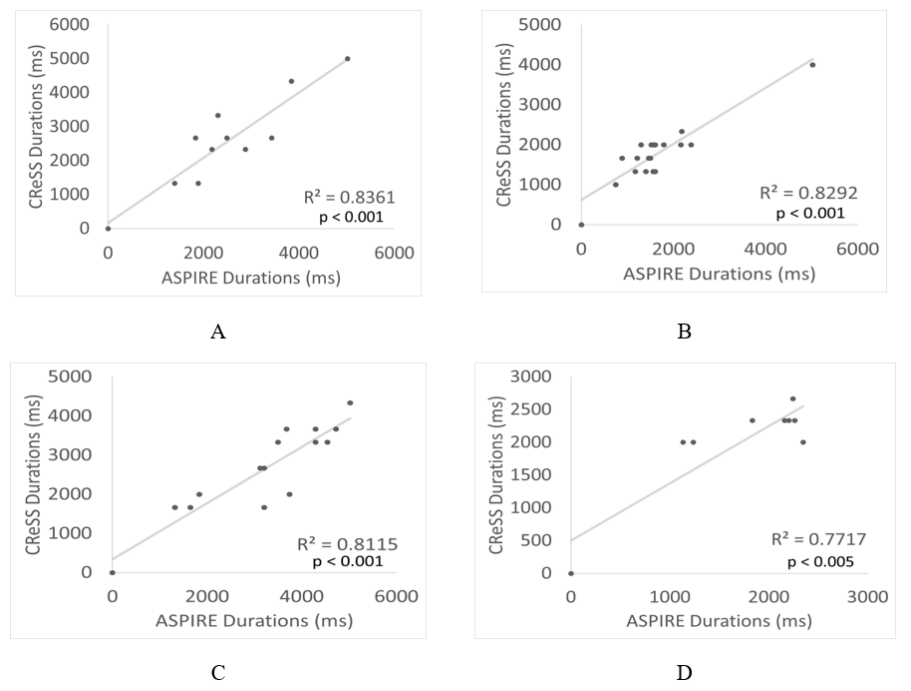
Correlations of individual puff durations collected via the Clinical Research Support System (CReSS) device and Automated Smoking PerceptIon and REcording (ASPIRE) for participants (A) P15, (B) P17, (C) P19, and (D) P8.

**Figure 9:**
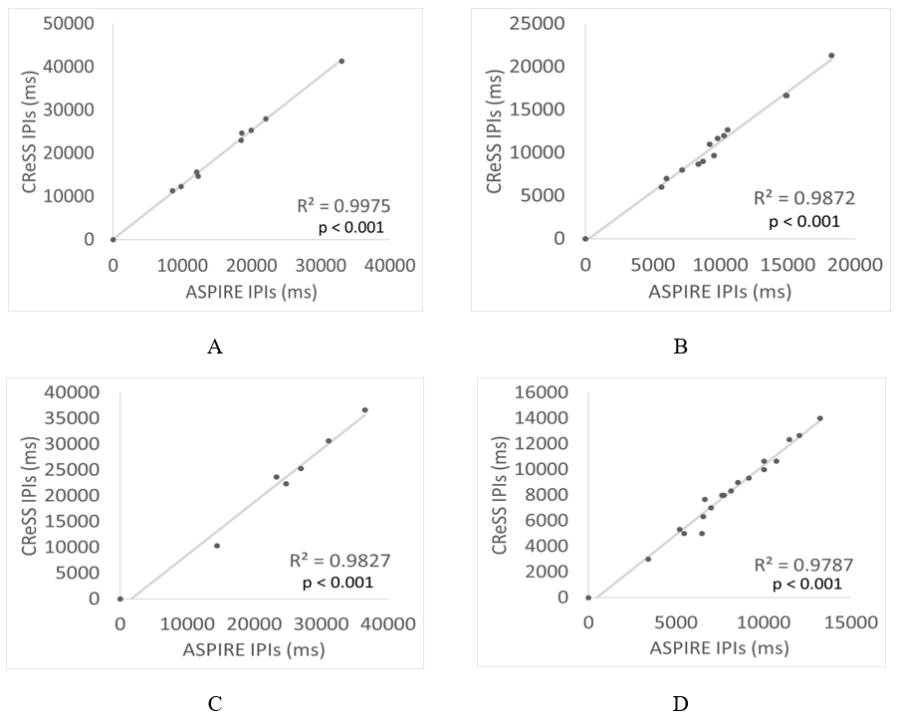
Correlations of individual interpuff intervals (IPIs) collected via the Clinical Research Support System (CReSS) device and Automated Smoking PerceptIon and REcording (ASPIRE) for participants (A) P15, (B) P19, (C) P7, and (D) P17.

Due to potential error in smoking topography data acquired by CReSS, the data are not usually used to perform detailed statistical analyses or inferences, and instead, the median values for PD and IPI are used. The developer of CReSS, Borgwaldt, recently released a method for correction of these errors, but it still remains widely variable in its success. However, in contrast to CReSS, detailed recording by ASPIRE allows for the meaningful study of mean, standard deviation, and other statistical moments of PD and IPI for each participant. Figure 7 illustrates the smoking topography for a representative participant (P2) reported by CReSS and ASPIRE. Both methods reported a total of 10 recorded puffs (illustrated in green bars) separated by 9 IPI intervals (illustrated in blue bars). Also, both methods reported similar values for the median PD and IPI. However, it is clear that the duration of the entire event is highly discrepant across the 2 methods. Furthermore, visual inspection of the smoking session reported by CReSS consists of only 8 decipherable puffs (green regions). This is due to very short IPIs of 5 milliseconds that render 2 puffs unseparated in the figure. On the other hand, the same smoking session reported by ASPIRE is well organized into the expected shorter puffs that are separated by longer IPIs. The trend marked as cCReSS in this figure corresponds to the corrected CReSS data by only correcting 5 elements (puffs or IPIs out of 20) of the smoking session. These were corrected by substituting the abnormally short or long IPIs with the average of the remaining IPIs in the CReSS data for the participant. The correlation between the CReSS and ASPIRE reported data improved from 0.05 to 0.80 after correcting for the discrepant data. Figure 8 and Figure 9 demonstrate other examples of similarity between the CReSS and ASPIRE data after correcting for outliers. These examples have R^2^ values in the ranges of 0.77-0.83 for PD and 0.97-0.99 for IPI. These correlation values indicate a similarity reported by the 2 different methods with statistical significance of *P*<.005.

## Discussion

### Principal Findings

Findings from this study provide direct evidence that ASPIRE accurately assesses multiple components of smoking behavior in controlled laboratory settings. Our study revealed 3 important findings. First, ASPIRE is highly effective in accurately detecting when a person initiates smoking and how many puffs of a cigarette is inhaled. Second, ASPIRE may be used to accurately characterize the duration of each puff—which may provide a dose indicator when used in conjunction with puff count. Finally, ASPIRE can detect time between each puff (IPI), which may provide a meaningful metric of episodic smoking compulsivity. These findings suggest that ASPIRE can be tested in naturalistic settings as a potential means to assess smoking behavior in daily life.

Smartwatches have the potential to significantly advance the study of human behavior in situ; however, the reliability of the information reported by wearable devices has been questioned. In this study, we investigated and demonstrated the reliability of the data reported by ASPIRE in the laboratory setting compared to the CReSS and visual recording of the smoking sessions. Moreover, though other technologies have been recently developed to detect smoking behavior at the puff or session level [8,14,19], this is the first study to demonstrate characterization of smoking topography (namely PD and IPI) as performed by ASPIRE.

The ability to passively collect and accurately characterize multiple components of smoking behavior in the natural environment is a critical step in monitoring smoking, characterizing smoking outcomes in outpatient clinical trials, and developing real-time adaptive interventions or personalizing smoking cessation interventions. Much of our knowledge about the mechanisms that elicit smoking behavior is obtained from observation of behavior in laboratory settings. For example, research has examined how exposure to smoking stimuli (for example, image of cigarette lighter) [29], acute stressors or mood inductions [eg, 30,31], fasting [30], and interventions [31,32] affect smoking behavior under controlled laboratory conditions. Upon validation of ASPIRE in naturalistic settings, ASPIRE can be used to examine whether laboratory-based findings generalize to real-world settings. In future studies, ASPIRE-detected smoking may be developed to incorporate randomly prompted app, text, or smartwatch surveys asking about precipitants of smoking behavior (eg, stress, craving, environmental contexts). Alternatively, several passive technologies can be combined. For example, measures of electrodermal activity or heart rate (ie, physiological arousal) recorded via mobile devices may be collected in addition to assessing the timing, count, and characteristics of cigarettes smoked in real-world settings. The combination of these active and passive measures will significantly improve fidelity for characterizing the factors that maintain smoking behavior among individuals and thus facilitate precision medicine and treating TUD.

In addition, real-time technology has the potential to greatly improve assessment of smoking outcomes in smoking cessation clinical trials [33]. The traditional outcome measure in smoking cessation clinical trials is biomarker-confirmed abstinence [34,35]. Typically, this is assessed via participant report of abstinence for a certain number of days (ie, 7-day abstinence) and confirmed in laboratory via carbon monoxide or cotinine. These outcomes are subject to errors in retrospective recall and intentional misreporting. ASPIRE can provide objective evidence of smoking while also indicating when the smartwatch is removed for the purposes of determining adherence with wearing the smartwatch. Remote technologies also help to extend the reach of clinical trials. Individuals with the necessary technology can participate in a smoking cessation trial remotely (ie, at a different location than the research team) while still providing rigorous evidence of smoking or abstinence.

Finally, the incorporation of AI and machine learning techniques to automatically detect and report smoking behavior can assist in the delivery of personalized and just-in-time interventions. AI algorithms can incorporate the precise information regarding the context and timing of cigarettes smoked gained from ASPIRE to determine when an individual is most likely to smoke. Interventions can be delivered pre-emptively to prevent smoking or relapse [eg, 38].

### Limitations

A number of limitations must be considered when interpreting the current findings. First, validation of ASPIRE requires comparison to a gold standard measure. However, CReSS, as the gold standard comparator used in this study, suffers its own limitations. The CReSS device identifies smoke topography only based on the inhalation patterns and does not incorporate any information regarding the exhalation activity. Therefore, the CReSS device may identify a single puff that is composed of numerous discontinuous puffs as multiple short puffs separated by short IPIs. For instance, in Figure 4, it was shown that CReSS recorded a significant number of IPIs of 1 second or less. While IPIs of this length are theoretically possible, their appearance in such an abundance reported by CReSS is highly suspect. The short IPIs reported by CReSS contribute to skewing the overall statistics presented, which lowered concordance between the CReSS puffs and ASPIRE-collected puffs. Based on the review of the visual recordings in this study, we have confirmed that the ASPIRE approach provides a more consistent and reproducible report of the smoking topography than the CReSS device. Furthermore, we have also demonstrated the consistency of smoking topography reported by smartwatches and the CReSS device in laboratory settings. Our results conclude that the PD and IPI reported by both devices exhibit a substantial degree of correlation after the exclusion of the outliers reported by the CReSS device.

A second limitation of this study is that the laboratory is an unnatural smoking environment that may elicit unnatural smoking behavior from the participants, including the use of CReSS to smoke. ASPIRE can record and report smoking behavior in natural settings, though it is possible that the accuracy of ASPIRE in the laboratory does not generalize to these settings or to cigarettes not smoked via CReSS. Thus, future efforts should examine the comparability of cigarettes smoked with and without CReSS and the applicability of ASPIRE in studying smoking behavior in natural settings.

### Comparison to Prior Work

Although there have been prior reports [8,9,12-14,18] of identifying smoking sessions using smartwatches, to our knowledge, there has been no other smoking topography work with which to compare these results. Our reported results constitute the first instance of comparing smoking data collected from smartwatches to smoking data collected from the industry-standard CReSS device.

### Conclusions

In summary, this study provides preliminary evidence of ASPIRE’s potential to accurately and reliably detect smoking characteristics passively and in real-time. The ability to observe smoking behavior in situ holds great promise in advancing research on the mechanisms that maintain cigarette smoking, measuring behavior change in the context of clinical trials, and the development of novel, real-time interventions for smoking cessation and just-in-time relapse prevention interventions.

## Abbreviations

AI: artificial intelligence
ASPIRE: Automated Smoking PerceptIon and REcording
CReSS: Clinical Research Support System
IPI: interpuff interval
PD: puff duration
TUD: Tobacco Usage Disease

## References

[1] Centers for Disease Control and Prevention (CDC) (2002). Annual smoking-attributable mortality, years of potential life lost, and economic costs--United States, 1995-1999. MMWR Morb Mortal Wkly Rep.

[2] Lloyd-Jones D, Adams RJ, Brown TM, Carnethon M, Dai S, De Simone G, Ferguson TB, Ford E, Furie K, Gillespie C, Go A, Greenlund K, Haase N, Hailpern S, Ho PM, Howard V, Kissela B, Kittner S, Lackland D, Lisabeth L, Marelli A, McDermott MM, Meigs J, Mozaffarian D, Mussolino M, Nichol G, Roger VL, Rosamond W, Sacco R, Sorlie P, Roger VL, Stafford R, Thom T, Wasserthiel-Smoller S, Wong ND, Wylie-Rosett J, American Heart Association Statistics Committee and Stroke Statistics Subcommittee (2010). Heart disease and stroke statistics--2010 update: a report from the American Heart Association. Circulation.

[3] Vinci C, Haslam A, Lam CY, Kumar S, Wetter DW (2018). The use of ambulatory assessment in smoking cessation. Addict Behav.

[4] Shoaib M, Bosch S, Scholten H, Havinga PJM, Incel OD (2015). Towards detection of bad habits by fusing smartphone and smartwatch sensors.

[5] Shoaib M, Bosch S, Incel O, Scholten H, Havinga P (2016). Complex Human Activity Recognition Using Smartphone and Wrist-Worn Motion Sensors. Sensors (Basel).

[6] Shaw RJ, Yang Q, Barnes A, Hatch D, Crowley MJ, Vorderstrasse A, Vaughn J, Diane A, Lewinski AA, Jiang M, Stevenson J, Steinberg D (2020). Self-monitoring diabetes with multiple mobile health devices. J Am Med Inform Assoc.

[7] Shaw RJ, Barnes A, Steinberg D, Vaughn J, Diane A, Levine E, Vorderstrasse A, Crowley MJ, Wood E, Hatch D, Lewinski A, Jiang M, Stevenson J, Yang Q (2019). Enhancing Diabetes Self-Management Through Collection and Visualization of Data From Multiple Mobile Health Technologies: Protocol for a Development and Feasibility Trial. JMIR Res Protoc.

[8] Skinner AL, Stone CJ, Doughty H, Munafò MR (2019). StopWatch: The Preliminary Evaluation of a Smartwatch-Based System for Passive Detection of Cigarette Smoking. Nicotine Tob Res.

[9] Cole CA, Janos B, Anshari D, Thrasher JF, Strayer S, Valafar H (2016). Recognition of Smoking Gesture Using Smart Watch Technology.

[10] Cole CA, Thrasher F, Strayer S, Valafar H (2017). Resolving Ambiguities in Accelerometer Data Due to Location of Sensor on Wrist in Application to Detection of Smoking Gesture.

[11] Imtiaz MH, Ramos-Garcia RI, Wattal S, Tiffany S, Sazonov E (2019). Wearable Sensors for Monitoring of Cigarette Smoking in Free-Living: A Systematic Review. Sensors (Basel).

[12] Senyurek V, Imtiaz M, Belsare P, Tiffany S, Sazonov E (2019). Cigarette Smoking Detection with An Inertial Sensor and A Smart Lighter. Sensors (Basel).

[13] Agac S, Shoaib M, Durmaz Incel O (2020). Smoking recognition with smartwatch sensors in different postures and impact of user’s height. AIS.

[14] Cole CA, Anshari D, Lambert V, Thrasher JF, Valafar H (2017). Detecting Smoking Events Using Accelerometer Data Collected Via Smartwatch Technology: Validation Study. JMIR Mhealth Uhealth.

[15] Parate A, Chiu MC, Chadowitz C, Ganesan D, Kalogerakis E (2014). RisQ: Recognizing Smoking Gestures with Inertial Sensors on a Wristband. MobiSys.

[16] Maurer U, Smailagic A, Siewiorek DP, Deisher M (2006). Activity Recognition and Monitoring Using Multiple Sensors on Different Body Positions.

[17] Sazonov E, Lopez-Meyer P, Tiffany S (2013). A wearable sensor system for monitoring cigarette smoking. J Stud Alcohol Drugs.

[18] Odhiambo CO, Chrisogonas AC, Torkjazi A, Valafar H (2019). State Transition Modeling of the Smoking Behavior using LSTM Recurrent Neural Networks.

[19] Dar R (2018). Effect of Real-Time Monitoring and Notification of Smoking Episodes on Smoking Reduction: A Pilot Study of a Novel Smoking Cessation App. Nicotine Tob Res.

[20] Akyazi O, Batmaz S, Kosucu B, Arnrich B (2017). SmokeWatch: A smartwatch smoking cessation assistant.

[21] Valafar H, Cole C, Thrasher J, Strayer S (2018). Wearable computing device featuring machine-learning-based smoking detection. United States Patent Application Publication.

[22] Shoaib H, Scholten PJM, Havinga OD (2016). A hierarchical lazy smoking detection algorithm using smartwatch sensors.

[23] Zacny JP, Stitzer ML, Brown FJ, Yingling JE, Griffiths RR (1987). Human cigarette smoking: effects of puff and inhalation parameters on smoke exposure. J Pharmacol Exp Ther.

[24] Roche DJ, Bujarski S, Hartwell E, Green R, Ray LA (2015). Combined varenicline and naltrexone treatment reduces smoking topography intensity in heavy-drinking smokers. Pharmacol Biochem Behav.

[25] Pickworth W, Lee E, Malson J, Moolchan ET, Waters A (2003). Smoking topography: Reliability and validity in dependent smokers. Nicotine & Tobacco Research.

[26] Blank MD, Disharoon S, Eissenberg T (2009). Comparison of methods for measurement of smoking behavior: mouthpiece-based computerized devices versus direct observation. Nicotine Tob Res.

[27] Perkins KA, Karelitz JL (2020). A Procedure to Standardize Puff Topography During Evaluations of Acute Tobacco or Electronic Cigarette Exposure. Nicotine Tob Res.

[28] Froeliger B, McConnell PA, Bell S, Sweitzer M, Kozink RV, Eichberg C, Hallyburton M, Kaiser N, Gray KM, McClernon FJ (2017). Association Between Baseline Corticothalamic-Mediated Inhibitory Control and Smoking Relapse Vulnerability. JAMA Psychiatry.

[29] Conklin CA, McClernon FJ, Vella EJ, Joyce CJ, Salkeld RP, Parzynski CS, Bennett L (2019). Combined Smoking Cues Enhance Reactivity and Predict Immediate Subsequent Smoking. Nicotine Tob Res.

[30] Leeman RF, O'Malley SS, White MA, McKee SA (2010). Nicotine and food deprivation decrease the ability to resist smoking. Psychopharmacology (Berl).

[31] McClure EA, Baker NL, Gray KM, Hood CO, Tomko RL, Carpenter MJ, Ramakrishnan VR, Buchanan CJ, Saladin ME (2020). The influence of gender and oxytocin on stress reactivity, cigarette craving, and smoking in a randomized, placebo-controlled laboratory relapse paradigm. Psychopharmacology (Berl).

[32] McKee SA, Weinberger AH, Shi J, Tetrault J, Coppola S (2012). Developing and validating a human laboratory model to screen medications for smoking cessation. Nicotine Tob Res.

[33] Tomko RL, McClure EA, Squeglia LM, Treloar Padovano H, McRae-Clark AL, Baker NL, Carpenter MJ, Gray KM (2019). Methods to reduce the incidence of false negative trial results in substance use treatment research. Curr Opin Psychol.

[34] Benowitz N, Bernert J, Foulds J, Hecht S, Jacob P, Jarvis MJ, Joseph A, Oncken C, Piper ME (2020). Biochemical Verification of Tobacco Use and Abstinence: 2019 Update. Nicotine Tob Res.

[35] Piper ME, Bullen C, Krishnan-Sarin S, Rigotti NA, Steinberg ML, Streck JM, Joseph AM (2020). Defining and Measuring Abstinence in Clinical Trials of Smoking Cessation Interventions: An Updated Review. Nicotine Tob Res.

